# Determination of Heavy Metal Residues in Slaughtered Camels at Sokoto and Gusau Modern Abattoirs, Nigeria

**DOI:** 10.5696/2156-9614-8.20.181204

**Published:** 2018-12-03

**Authors:** Akawu Bala, Abdulkadir Usman Junaidu, Mohammed Danlami Salihu, Bello Mohammed Agaie, Mahmud Abdulahi Saulawa, Aliyu Ibrahim Musawa, Kabir H. Ahmad

**Affiliations:** 1 National Veterinary Research Institute, Vom, Plateau State, Nigeria; 2 Faculty of Veterinary Medicine, Usmanu Danfodiyo University, Sokoto, Nigeria; 3 Veterinary Council of Nigeria, Maitama, Abuja, Nigeria; 4 Faculty of Veterinary Medicine, Ahmadu Bello University, Zaria, Nigeria

**Keywords:** abattoir, camels, kidney, liver, muscle, hide, blood

## Abstract

**Background.:**

Heavy metals can pose health risks to both animals and humans. Objectives. To determine the concentrations of lead (Pb), cadmium (Cd), and chromium (Cr) in samples taken from the kidney, liver, muscle, hide, and blood of camels slaughtered at both Sokoto and Gusau modern abattoirs.

**Methods.:**

The concentrations of Pb, Cd, and Cr in tissues and organs of camels slaughtered at both Sokoto and Gusau modern abattoirs were determined using atomic absorption spectrophotometry. A total of 120 samples were collected.

**Results.:**

All the samples collected tested positive for Pb, Cd and Cr. The overall mean concentrations of Pb, Cd and Cr in tissues and organs of slaughtered camels at Sokoto modern abattoir ranged from 0.11 mg/kg to 0.35 mg/kg, 0.05 to 0.8 mg/kg and 0.41 to 0.59 mg/kg, respectively, while at Gusau modern abattoir, the overall mean concentrations of Pb, Cd and Cr ranged from 0.20 mg/kg to 1.17 mg/kg, 0.01 to 0.14 mg/kg and 0.13 to 0.51 mg/kg, respectively.

**Discussion.:**

The concentration of Pb in the tissues and organs of camels slaughtered at Gusau modern abattoir was high compared to in camels slaughtered at Sokoto modern abattoir, while the concentrations of Cd and Cr in the tissues and organs of camels slaughtered at Sokoto modern abattoir were high compared to those in tissues and organs of camels slaughtered at Gusau modern abattoir. There were significant differences (P<0.05) in the concentration of Pb, Cd, and Cr in samples taken from the kidney, liver, muscle, hide, and blood of slaughtered camels at both Sokoto and Gusau.

**Conclusions.:**

Camels slaughtered at both Sokoto and Gusau modern abattoirs were exposed to Pb, Cd, and Cr.

The tissues and organs of camels slaughtered at both Sokoto and Gusau contain Pb, Cd, and Cr. Prolonged exposure through consumption of these tissues and organs should be avoided.

**Competing Interests.:**

The authors declare no competing financial interests.

## Introduction

Environmental contamination with toxic heavy metals has important public health implications as heavy metals can bio-accumulate in the environment and pose health risks. The presence of heavy metals in the tissues and organs of animals and humans may be responsible for many diseases, especially cardiovascular, renal, nervous and bone disorders.^1^,^2^ Some heavy metals are considered to be carcinogenic, mutagenic and teratogenic.^3^

Indiscriminate dumping of waste materials on land and into water bodies, illegal mining of ores and many other human activities that result in waste generation are common in Nigeria, and some of these waste materials may contain heavy metals that are toxic to both humans and animals.^4^,^5^ Cattle, camels, goats and other ruminants graze freely and drink water from ponds, streams, rivers and other possibly contaminated water sources and these metals may bioaccumulate in their tissues and organs. The meat and meat products from these animals reach market without monitoring of the concentration of these toxic heavy metals. Therefore, the present study aims to determine the presence of lead (Pb), cadmium (Cd) and chromium (Cr) in samples taken from the kidney, liver, muscle, hide and blood of slaughtered camels at Sokoto and Gusau modern abattoirs. It also seeks to evaluate the concentrations of these metals in selected organs and tissues in order to determine whether the concentrations of these metals are within the permissible concentrations recommended by the Food and Agricultural Organization (FAO), World Health Organization (WHO), and the United States Department of Agriculture (USDA) for human consumption.

## Methods

Sokoto modern abattoir is located in the Sokoto North Local Government Area of Sokoto State, Nigeria. Sokoto State is located in northwestern Nigeria between longitudes 4°8′ E and 6°54′ E and latitudes 12° N and 13°58′ N. The state shares boundaries with the Niger Republic to the north, Kebbi State to the west and Zamfara State to the east. Sokoto State covers a total land area of about 32,000 km^2^ with an estimated human population of 3,702,676.^6^ The state ranks second in terms of livestock population in Nigeria with an estimated 3 million cattle, 3 million sheep, 5 million goats, 4,600 camels, 52,000 donkeys and a number of other local and exotic poultry species.^7^,^8^

Gusau modern abattoir is located along Gusau-Sokoto road on the outskirts of Gusau town, opposite the Nigeria National Petroleum Corporation depot. Gusau is the state capital of Zamfara State. Zamfara State is located at latitude 11°10′ N and longitude 6°15′ E, covering an area of 39,762 km^2^ with an estimated human population of 3,582,912.^6^,^9^ The climate is semi-arid, with average temperatures above 28.5°C, annual rainfall of less than 1000 mm and relative humidity below 70%.^10^ It shares borders with Kebbi, Kaduna, Sokoto, Niger and Katsina States. It also shares an international boundary with the Niger Republic to the north.^9^ The majority of the population is engaged in farming and livestock rearing. The state has an estimated livestock population of 3,190,010 cattle, 4,933,304 sheep, 5,177,348 goats, 34,796 camels, and host of other local and exotic poultry species.^7^,^8^ Gusau abattoir is the main abattoir in the state. Cattle, sheep, goats and camels are then slaughtered for human consumption.

### Study design

The present study was cross-sectional in design. Samples were collected on a weekly basis using a simple convenience sampling method until the desired numbers of samples were collected. Samples were collected from Sokoto and Gusau modern abattoirs at the same time of year. The samples consisted of kidney, liver, muscle, hide and blood of 15 slaughtered camels, selected by convenience in each of the abattoirs.

In each abattoir (Sokoto, and Gusau); sixty samples (60) comprised of 12 livers, kidneys, hides, and muscle and blood samples were collected from slaughtered camels. About 100 g of liver from any lobe, a whole kidney (either right or left), about 100 g of any part of the muscle, approximately 100 g of any part of singed or un-singed hide, and about 100 ml of blood from slaughtered camels were purchased. Each of the samples was packed in a sterile polythene bag properly labeled with permanent marker and was transported to the Veterinary Public Health and Preventive Medicine Laboratory of the Faculty of Veterinary Medicine, Usmanu Danfodiyo University, Sokoto, where samples were frozen and stored.

All of the frozen samples were packed on ice blocks in coolers and transported to the National Research Institute for Chemical Technology in Zaria, Kaduna State, Nigeria for further processing and analysis.

Liver, kidney, muscle, hide, and blood samples were dried at 45°C using an oven. After drying, individual samples were crushed into fine powder using a mortar and pestle, and then 1.0 g of the fine powder sample was weighed out into a porcelain crucible. The crucible plus the fine powdered samples were ignited in a muffle furnace at 500°C for eight hours. The samples were then removed from the furnace and allowed to cool in desiccators and weighed again. The difference between the weight of the crucible plus ash and the weight of the crucible alone was used to calculate the percentage ash content of the sample. Then 5 cm^3^ of 1 M trioxonitrate (v) acid solution was added to the leftover ash and evaporated to dryness on a hot plate and returned to the furnace and heated again at 400°C for 15–20 minutes until a grayish-white ash was obtained. The samples were allowed to cool in desiccators, followed by the addition of 15 cm^3^ of 1 M hydrochloric acid to dissolve the ash and the solution was then filtered into 100 cm^3^ volumetric flasks. Distilled water was added to the flask to bring the volume up to 100 cm^3^.

In the prepared samples, Cd, Pb, and Cr residues were determined under specified conditions according to the manufacturer (AA-6800, Shimadzu atomic absorption spectrophotometer).^11^

Data are presented in tables and bar charts. One-way analysis of variance was used to establish significant differences between mean concentrations of Pb, Cd and Cr present in the kidney, liver, muscle, hide and blood at 95% confidence limits using Graphpad Instat 3.10 software for Windows 7.

## Results

The findings from the present study demonstrated a 100% prevalence rate of Pb, Cd and Cr in camels slaughtered at both Sokoto and Gusau modern abattoirs.

At Sokoto modern abattoir, the overall mean concentrations of Pb, Cd and Cr ranged from 0.11 mg/kg to 0.35 mg/kg, 0.05 to 0.8 mg/kg and 0.41 to 0.59 mg/kg, respectively (*Table 1*).

**Table 1:**
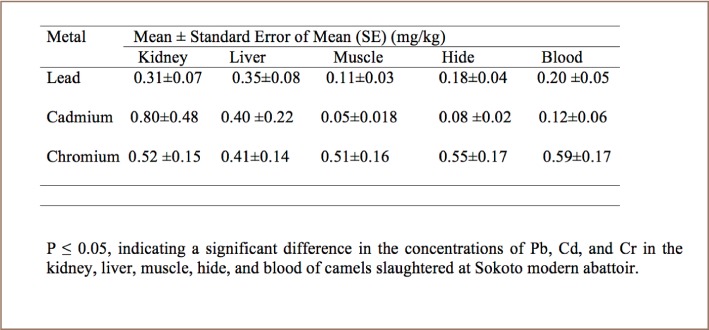
Mean and Standard Error (mg/kg) of Lead, Cadmium, and Chromium (mg/kg) in the Kidney, Liver, Muscle, Hide and Blood of Camels Slaughtered at Sokoto Modern Abattoir

At Gusau modern abattoir, the overall mean concentration of Pb, Cd and Cr ranged from 0.20 to 1.17 mg/kg, 0.01 to 0.14 mg/kg and 0.13 to 0.51 mg/kg, respectively (*Table 2*).

**Table 2:**
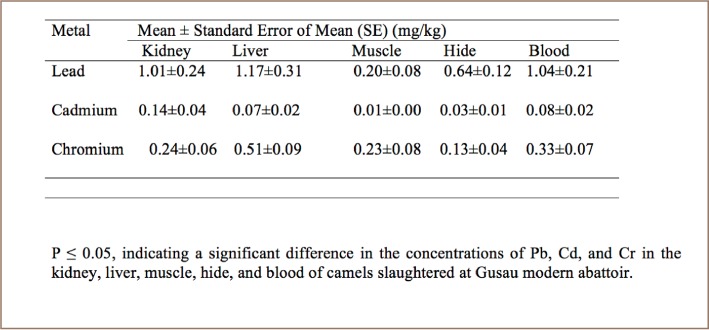
Mean and Standard Error (mg/kg) of Lead, Cadmium, and Chromium (mg/kg) in the Kidney, Liver, Muscle, Hide and Blood of Camels Slaughtered at Gusau Modern Abattoir

**Figure 1:**
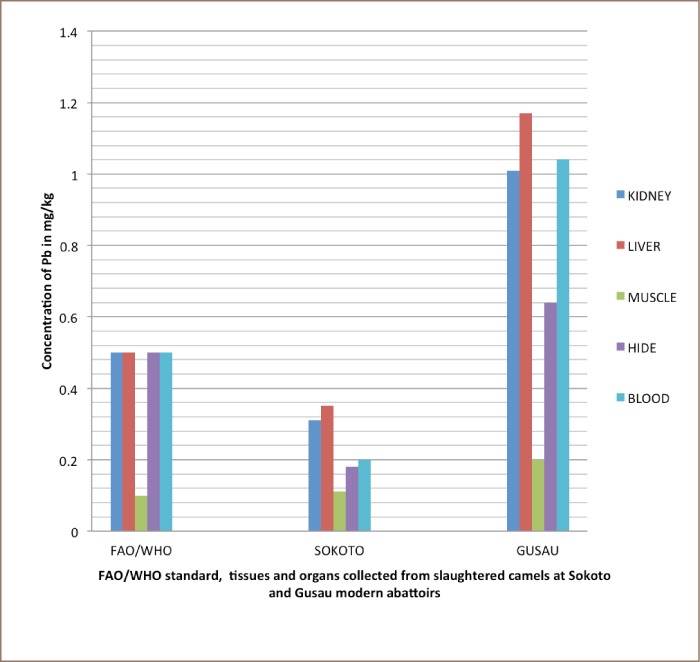
Bar charts showing mean concentration of Pb in kidney, liver, muscle, hide, and blood of slaughtered camels at Sokoto and Gusau modern abattoirs against FAO/WHO permissible concentrations

**Figure 2:**
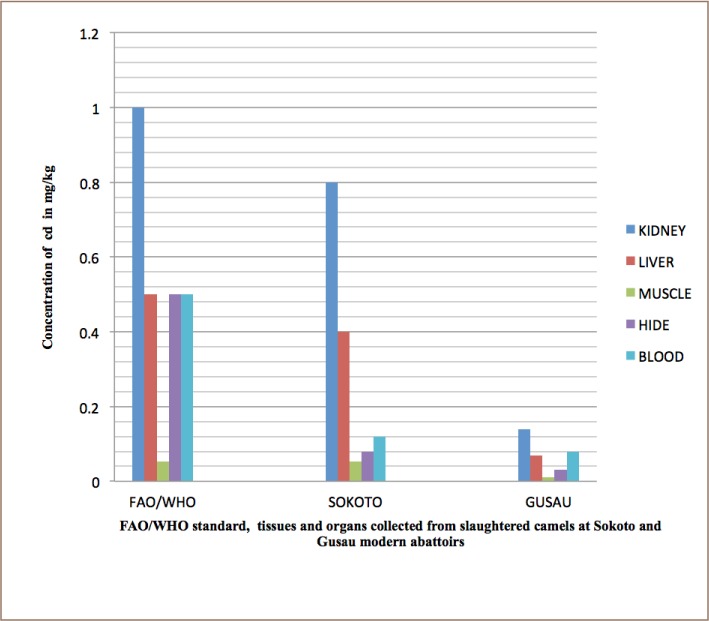
Bar charts showing mean concentration of Cd in kidney, liver, muscle, hide, and blood of slaughtered camels at Sokoto and Gusau modern abattoirs against FAO/WHO permissible concentrations.

**Figure 3:**
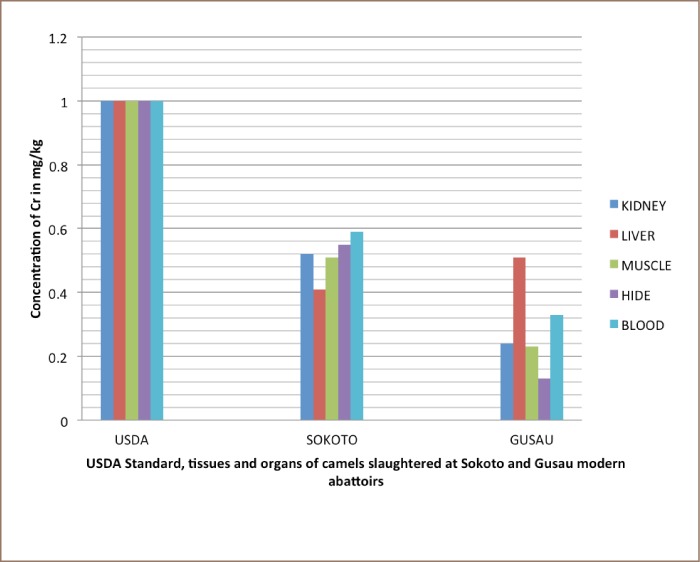
Bar charts showing mean concentration of Cr in kidney, liver, muscle, hide, and blood of slaughtered camels at Sokoto and Gusau modern abattoirs against USDA permissible concentration

Liver samples contained high concentrations of Pb compared to kidney, muscle, hide and blood samples (*Tables 3 and 4*). In addition, the concentration of Cd in kidney samples was high compared to in liver, muscle, hide and blood (*Tables 3 and 4, and Figures 1–3*) also see figures below.

**Table 3:**
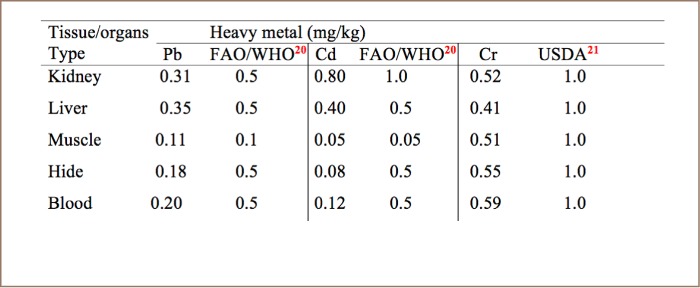
Mean Concentrations (mg/kg) of Lead, Cadmium, and Chromium Compared to International Standards in Selected Organs and Tissues of Camels Slaughtered at Sokoto Modern Abattoir

**Table 4:**
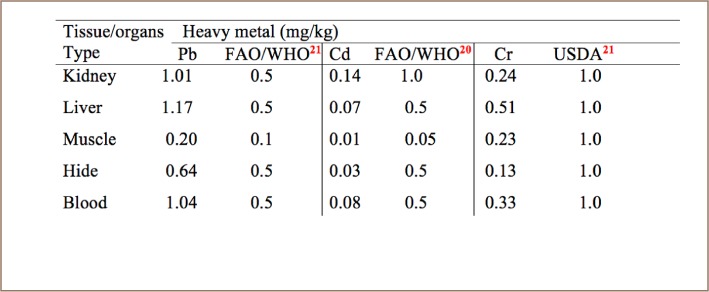
Mean Concentrations (mg/kg) of Lead, Cadmium, and Chromium Compared to International Standards in Selected Organs and Tissues of Slaughtered Camels at Gusau Modern Abattoir

## Discussion

The prevalence rate obtained in the present study was in agreement with the reported 100% prevalence of Pb and Cd in muscle and edible tissue of cattle slaughtered at a slaughter slab in Ibadan.^12^ However, the prevalence in the present study was high compared to the rates of 93% and 21.3% reported in southeastern and southwestern Nigeria, respectively.^13^,^14^ The variation in these reported prevalence may be a result of mining activities, household waste and other industrial sources of pollution which contaminate the environment in different geographical areas. Animals slaughtered at both Sokoto and Gusau modern abattoirs were purchased from Sokoto, Kebbi, and Zamfara States, other neighboring states and across international borders. Zamfara State experienced a well-known outbreak of lead poisoning in 2010 due to mining activity.^15^ Ruminants are bio-indicators for environmental contamination with heavy metals and therefore increases in the concentration of heavy metals in the environment can result in high prevalence in these animals.^16^ Contamination can also occur during processing of meat and meat products in the abattoirs due to the use of contaminated water, equipment or contaminated dust particles.^17^,^18^

The concentrations of Pb and Cd in slaughtered camels at both Sokoto and Gusau modern abattoirs in the present study were low compared to a study in Zamfara State, Nigeria, where Pb concentrations were 98.38 mg/kg in camel meat and 71.76 mg/kg in mutton, and Cd concentrations were 21.43 mg/kg in beef and 5.88 mg/kg in mutton.^19^ This variation may be a result of differences in exposures of the animals to heavy metals, along with differences in where the animals were reared before they were slaughtered.

The presence of heavy metals (Pb, Cd, and Cr) in the tissues and organs of camels slaughtered at both Sokoto and Gusau modern abattoir indicates that these animals were exposed to these heavy metals either through their feed, water, or inhalation in the environment where they were reared before slaughter for human and animal consumption.

Consumption of tissues and organs of camels slaughtered in these areas over a long period of time should be avoided in order to reduce the risk of developing health problems associated lead, cadmium and chromium.^20^,^21^

## Conclusions

The results of the present study show that camels slaughtered at both Sokoto and Gusau modern abattoirs have been exposed to Pb, Cd, and Cr.

Tissues and organs of camels slaughtered at both Sokoto and Gusau modern abattoirs contain Pb, Cd, and Cr prolonged exposure through consumption should be avoided in order to minimize the risk of developing health problems associated with these metals.
